# Oep23 forms an ion channel in the chloroplast outer envelope

**DOI:** 10.1186/s12870-015-0445-1

**Published:** 2015-02-12

**Authors:** Tom Alexander Goetze, Manali Patil, Ingrid Jeshen, Bettina Bölter, Sabine Grahl, Jürgen Soll

**Affiliations:** Department Biologie 1, Botanik, Ludwig-Maximilians-Universität München, Großhaderner Str. 2-4, 82152 Planegg-Martinsried, Germany; The Munich Center of Integrated Protein Science CIPSM, Ludwig-Maximilians-Universität München, Feodor-Lynen-Str. 25, 81377 München, Germany; Nanion Technologies GmbH, Gabrielenstr. 9, 80636 München, Germany

**Keywords:** Chloroplasts, Organelles, Ion channel, Membrane protein, Electrophysiology

## Abstract

**Background:**

Metabolite, ion and protein translocation into chloroplasts occurs across two membranes, the inner and the outer envelope. Solute and metabolite channels fulfill very important functions in integrating the organelles into the metabolic network of the cell. However so far only a few have been identified. Here we describe the identification and the characterization of the outer envelope protein of 23 kDa, Oep23 from garden pea.

**Results:**

Oep23 is found in the entire plant lineage from green algae to flowering plants. It is expressed in all organs and developmental states tested so far. The reconstituted recombinant protein Oep23 from pea forms a high conductance ion channel with a maximal conductance in the fully open state of 466 ± 14pS at a holding potential of +100 mV (in 250 mM KCl). The Oep23 channel is cation selective (P_K+_ : P_Cl-_ = 15 : 1) with a voltage dependent open probability of maximal V_mem_ = 0 mV.

**Conclusion:**

The data indicate that the Oep23 activity represents a single channel unit and does not assemble into a multiple pore complex like bacterial type porins or mitochondrial voltage dependent anion channel. Thus, Oep23 represents a new member of ion channels in the outer envelope of chloroplasts involved in solute exchange.

**Electronic supplementary material:**

The online version of this article (doi:10.1186/s12870-015-0445-1) contains supplementary material, which is available to authorized users.

## Background

All eukaryotic organisms represent chimera from the fusion of free living independent organisms. In a process named endosymbiosis, an ancient host cell took up an α-proteobacterium in a process most likely driven by the advantage of obtaining greater metabolic flexibility in a variable and changing environment. The transformation from a bacterial symbiont to an endosymbiotic organelle was accompanied by a loss of cellular autonomy due to a massive gene transfer from the endosymbiont to the host cell. Finally the endosymbiotic α-proteobacterium was converted into an organelle, which we know today as mitochondrion. This was followed by a second endosymbiotic event: the cell merging with an ancestral photosynthetic cyanobacterium, which gave rise to chloroplasts. Due to their gram-negative like bacterial ancestors, both mitochondria and chloroplasts are surrounded by two membranes, the inner and outer organellar membrane or envelope (for review see [1-5]). The inner membrane of mitochondria and chloroplasts corresponds to the plasma membrane of the bacterial endosymbiont. But also the outer membrane shows typical remnants of bacterial origin. Specifically the outer envelope of chloroplasts contains galactolipids and carotenoids found only in chloroplasts and cyanobacteria [6]. Phosphatidylcholine (PC), a typical eukaryotic lipid, is present only in the outer leaflet of the outer envelope [7]. Proteins in the outer membrane of gram-negative bacteria traverse the membrane only with ß-sheets and not by hydrophobic α-helices [8]. Many of the bacterial outer membrane proteins have a ß-barrel type structure forming so called porins, which form membrane channels for the uptake of ions and nutrients [9,10]. Likewise, the outer membrane of mitochondria and chloroplasts contain ß-barrel type ion channels of bacterial origin, most prominently the protein import channels Toc75 in chloroplasts and Sam50 in mitochondria, which are phylogenetically related to Omp85 [11,12]. Further porin-like channels have been characterized in the past, e.g. voltage dependent anion channel (VDAC) in mitochondria [13,14] or the chloroplast outer envelope proteins (Oep) 21, 24, and 37, which also form high conductance ß-barrel type ion channels [15-17]. On the other hand, typical host derived membrane components like PC in the outer leaflet of the chloroplast envelope or hydrophobic α-helical membrane proteins like Oep16 indicate that the outer organellar membranes today represent a mixture of host and endosymbiont derived building blocks [7,18].

The inner and outer chloroplast membranes delimit the organelle from the cytosol. They not only play key roles in the exchange of solutes and metabolites [19], but house numerous enzymatic activities, e.g. for the synthesis of lipids and pigments [20]. Their biochemical capacity as well as their role in intracellular communication and metabolic networking is therefore of prime interest and great physiological relevance. Proteomic studies have identified about 700 polypeptides in the chloroplast envelope membranes in both C3 and C4 plants [21-24]. Many of these proteins have still an unknown function and are poorly characterized. In recent years we have described a number of Oeps sharing several characteristics, which can be used to select them from a pool of unknown proteins for further studies. These features are:

i) lack of a predicted chloroplast targeting signal (most Oeps are targeted to plastids by non-classical internal peptide information) [23];
ii) high content of hydrophilic amino acids, because most channels have a ß-barrel type conformation and not hydrophobic α-helices;
iii) neutral or alkaline isoelectric point, like VDAC, OmpF, Oep21, Oep24 or Oep37;
iv) lack of predicted hydrophobic transmembrane helices.

When we combined these assumptions with published data [21-24] as well as our own proteomic data from purified outer envelopes of pea chloroplasts (Soll, unpublished) we were able to select several polypeptides for further studies, one of which is described here as Oep23.

## Results and discussion

### Identification of Oep23

The peptide sequence obtained in our own proteomic analysis from purified outer envelopes of pea chloroplasts matched to a number of EST pea contigs [25], which in combination with the identified sequence of the Arabidopsis homolog (At2g17695) could be used for the isolation and sequencing of a complete cDNA sequence from pea (GenBank accession No KJ939359, Additional file 1: Figure S2). PsOep23 has a calculated molecular weight of 23.6 kDa and an isoelectric point of 9.0. Oep23 homologs have been detected in the chloroplast proteome of Arabidopsis [21,24], pea [22] and maize [22,24]. Proteomic studies of chloroplast subfractions localized it further to the envelope membranes from Arabidopsis [21], pea [22] and maize [22, accession No AC217840.3_FGT00]. Neither a chloroplastic targeting signal nor hydrophobic peptide sequence could be detected, which is in line with the observation made by Zybailov *et al.* [23], who identified many plastidic proteins without predicted pre-sequence. A large part of Oep23, i.e. aa 48–198 in Arabidopsis, is annotated as domain of unknown function 1990 (DUF1990, Pfam 09348). This domain can be found in many bacterial proteins, but barely in eukaryotes, except the plant lineage (Additional file 1: Figure S2). In soybean Oep23 (Glyma16g22910) is expressed ubiquitously in all tissues indicating a general involvement in chloroplast metabolism (www.genevestigator.com/gv/plant.jsp). The Arabidopsis homolog At2g17695 is not represented on the complete genome cDNA microarray and can therefore not be assessed by publically available databanks. However, the protein has been detected in cotyledons, flowers, leaves and seeds (fgcz-atproteome.unizh.ch).

### Conductance and gating behavior of PsOep23

**Figure 1:**
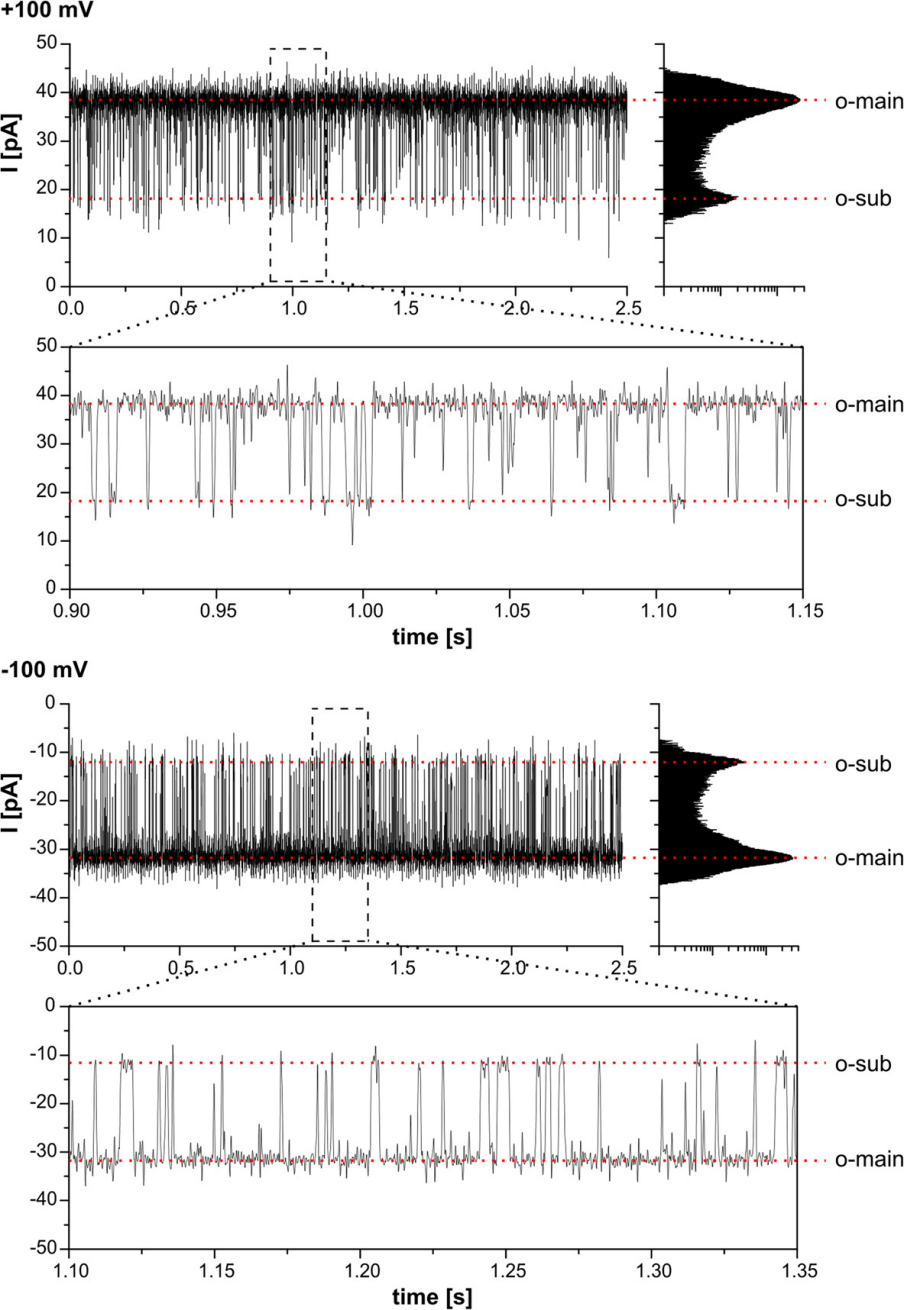
**PsOep23 shows channel activity.** Representative current traces of a bilayer containing a single active PsOep23 channel. Holding potential is set to +100 and −100 mV, respectively. The channel activity is characterized by short gating events from the open (o-main) to the sub-conductance state (o-sub). Some events are faster than the time resolution of the measurement which becomes apparent in the current histogram and a zoom into the trace. The electrolyte solution contained 250 mM KCl, 50 mM Mops/Tris pH 7.0 (symmetrical cis/trans).

**Figure 2:**
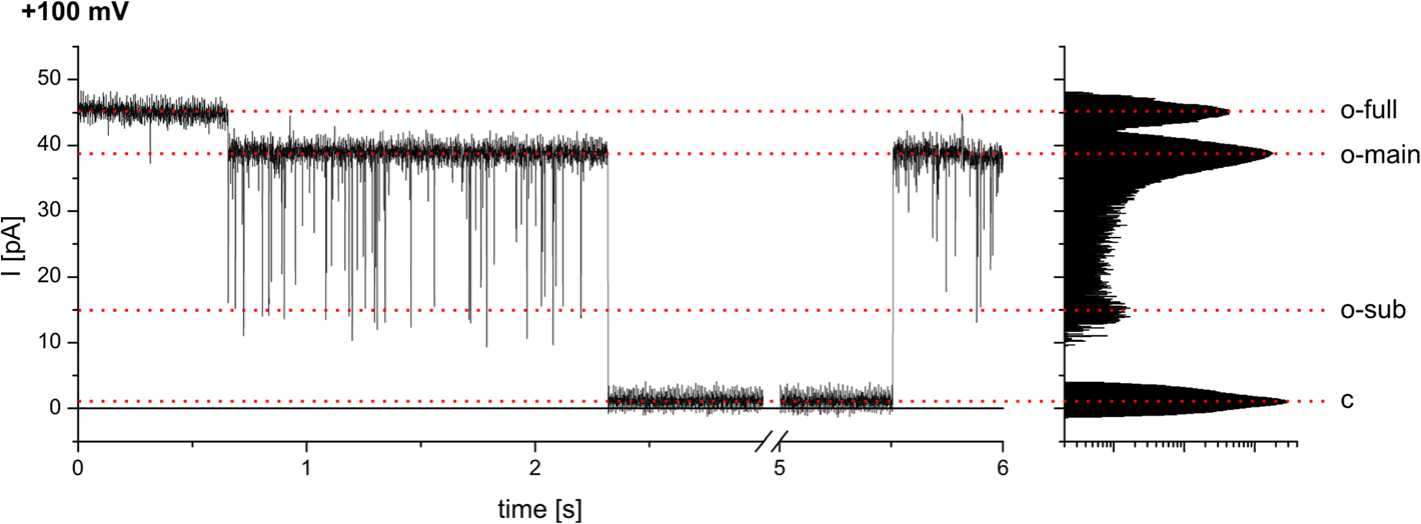
**PsOep23 temporarily fully opens.** Current trace of a bilayer containing a single active Oep23 channel at a holding potential of +100 mV. Apart from the main open (o-main) and the sub-conductance state (o-sub) the channel sometimes switched to its fully open state (o-full) and especially at holding potentials above +/−100 mV to the closed state (c). Electrolyte solution 250 mM KCl, 50 mM Mops/Tris pH 7.0 (symmetrical cis/trans).

After addition of reconstituted Oep23 to the bilayer chamber, a relatively large and characteristic channel activity with several sub-conductance states was observed (Figure 1). The channel had two main conductance states (o-main) of 382 ± 16 pS and 315 ± 15 pS at +100 and −100 mV, respectively (in 250 mM KCl, 10 or 50 mM Mops/Tris pH 7.0). In some experiments (about 15%) the channel exceeded the main conductance state, switching presumably to its fully open state (o-full) of 466 ± 14 pS and 424 ± 9 pS at +100 and −100 mV, respectively (Figure 2).

The gating behavior was characterized by frequent gating from either the main or the fully open state to a sub-conductance state (o-sub) of 185 ± 45 pS and 128 ± 18 pS at +100 and −100 mV, respectively. The sub-conductance state might be divisible into several sub-states, but this was not further analyzed in detail. Many of the gating events were shorter than the sampling interval of 100 μs (10 kHz), therefore were not resolved in time and thus only appeared as spikes in the current trace (Figure 1). The gating frequency fell into two modes i.e. a rapid gating activity with an average of 51 ± 25 events/s and a ~10 times less frequent gating activity of 6 ± 3 events/s. These two different gating modes could not be assigned to a specific open state of the channel, but instead were observed at the main (o-main) and the fully open state (o-full). At holding potentials below ±100 mV complete closure events (c) were rarely observed and happened in a stochastic manner. The time the channel spent in the closed state could last from a few milliseconds up to even seconds (Figure 2 and below). At holding potentials above ±100 mV the probability for a complete closure increased (data not shown).

**Figure 3:**
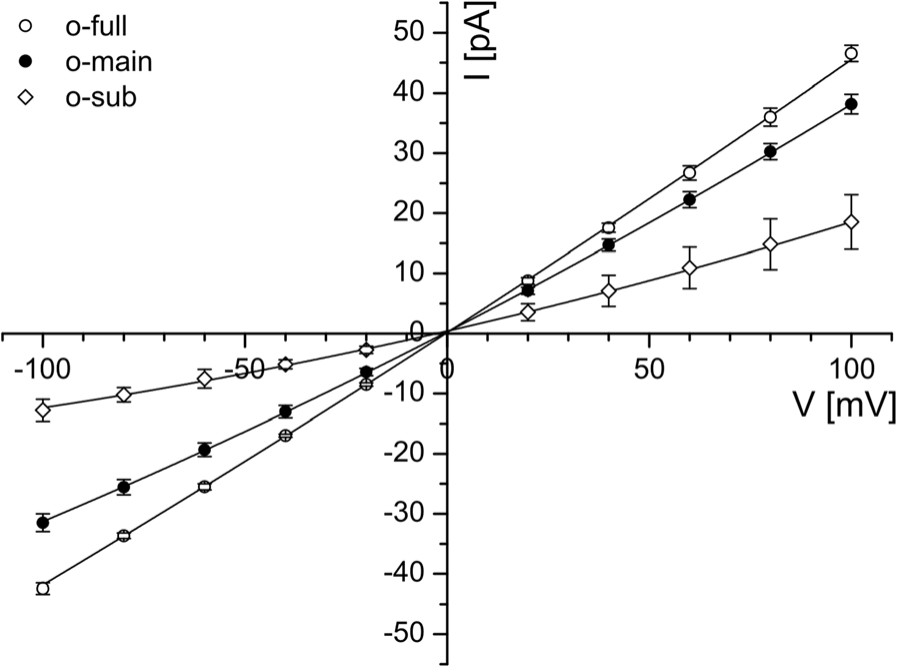
**Verification of PsOep23 orientation in a lipid bilayer.** The I/V relationship revealed a slightly rectifying current of all open states. This rectification helps to identify the orientation of the channel after fusion with the bilayer, which was randomly distributed. For clarity the large conductance was always allocated to positive holding potentials. The data points represent the average of 16 independent experiments. Electrolyte solution 250 mM KCl, 50 mM Mops/Tris pH 7.0 (symmetrical cis/trans).

The channel had slightly rectifying properties, i.e. the conductance at −100 mV reached only about 70-90% of the value at +100 mV (Figure 3). On the basis of this rectification it became obvious that the channel inserted in a random orientation into the bilayer. For the analysis and the generation of the I/V curve data from 16 independent bilayers were accumulated in the way that the large conductance side was allocated to positive holding potentials.

In all experiments complete closure events happened instantaneously in one step and not in e.g. three sequential steps, which would be typical for trimeric beta-barrel proteins like OmpF [26]. On the basis of this observation we assume that the PsOep23 activity is a single channel unit and does not assemble into a multiple pore complex in our conditions. The conductance of the fully opened PsOep23 is in the range of other Oep proteins [15-18] studied with a similar approach and in the same electrolyte conditions.

### Ion selectivity of PsOep23

**Figure 4:**
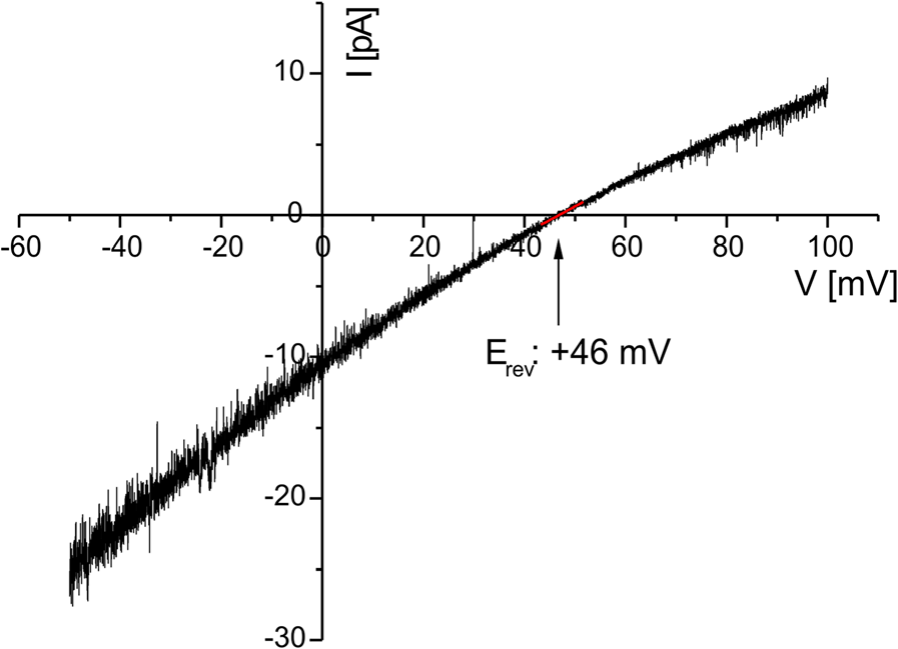
**PsOep23 is cation selective.** Current response of a voltage ramp from −50 to +100 mV (voltage slope: 7.5 mV/s) in asymmetric electrolyte conditions (cis/trans 20/250 mM KCl). Oep23 has a pronounced cation selectivity. The reversal potential (E_rev_) of +46 mV translates into a permeability ratio of K^+^: Cl^−^ of 15 : 1 by using the GHK equation and activities instead of concentrations.

The channel displayed a pronounced cation selectivity with a reversal potential (E_rev_) of +46 mV in asymmetric electrolyte conditions (cis/trans 20/250 mM KCl). Considering the activity coefficients of KCl this translates into a relative permeability ratio of K^+^ and Cl^−^ of 15 : 1 by using the Goldmann-Hodgkin-Katz (GHK) equation (Figure 4).

To test the selectivity of PsOep23 for different cation species we chose NaCl and KCl electrolyte concentrations (i.e. 242 mM NaCl and 250 mM KCl, respectively), which correspond to an activity of 175 mM (for further information see Methods). Due to the lower diffusion coefficient of Na^+^ ions in water, the bulk conductivity of 242 mM NaCl reaches only 80% compared to the value of 250 mM KCl. With no preference for any cation species we would have expected a reduced channel conductance of 80% in symmetric 242 mM NaCl in comparison to 250 mM KCl. In fact the conductance of PsOep23 in NaCl was even more reduced to 57 ± 4%, indicating a preference for K^+^ over Na^+^ ions. In addition, in bi-ionic conditions of cis 242 mM NaCl and trans 250 mM KCl, a reversal potential of E_rev_ +10.5 mV was measured. By using the GHK equation and keeping the permeability coefficients of K^+^ and Cl^−^ constant, this corresponds to a permeability relation of K^+^ : Na^+^ : Cl^−^ of 15 : 9 : 1 (or normalized to K^+^ permeability; 100% : 60% : 7%). So the two independent approaches show a comparable preference of PsOep23 for K^+^ ions over Na^+^ ions. The relatively large conductance and pronounced, but not absolute cation selectivity is a typical feature of wide and water-filled pores, which are found in the outer membranes of bacteria and organelles of endosymbiotic origin [17,27,28].

### Effect of added compounds

To further investigate the physiological role of PsOep23 in the chloroplast membrane, we tested the influence of various compounds on the current of the channel. Control conditions were 250 mM KCl, 50 mM Mops/Tris pH 7.0. To ensure that a possible change was due to the compound itself and not to any indirect side-effect we checked the pH and the bulk conductivity of the electrolyte before and after addition of the corresponding compound. Negatively charged compounds like malic acid or oxalacetate had no effect on the channel conductance, as it was also the case for adenine and thymine. The effects of the added compounds on channel activity are outlined in (Table 1).

**Table 1:** **Summary of effects on PsOep23 channel activity by the added compounds**

			**Change of**		
**Compound**	**Charge at pH 7**	**Conc. [mM]**	**Channel conductance**		**P**_**open**_
			**+100 mV**	**−100 mV**	
Malic acid	−2	20	-	-	-
Oxaloacetic acid	−2	2.5	-	-	-
Adenine	0	3-5	-	-	-
Thymine	+1	3-5	-	-	-
Ornithine	+1	2-10	-	-	-
Caderverine	+2	5-10	-	-	-
Spermidine	+3	5	−25%	−12%	-
		10	−36%	−21%	-
Spermine	+4	5	−28%	-	Yes
		10	−51%	−22%	Yes

Only spermidine and spermine at 5-10 mM induced a substantial reduction of single channel conductance while spermine alone also reduced the open probability. The bulk conductivity and pH were changed by max. 3% or 0.2 units, respectively (exception: thymine and adenine at 5 mM +0.7 pH units).

- : less than 10% charge.

P_open_: open probability.

**Figure 5:**
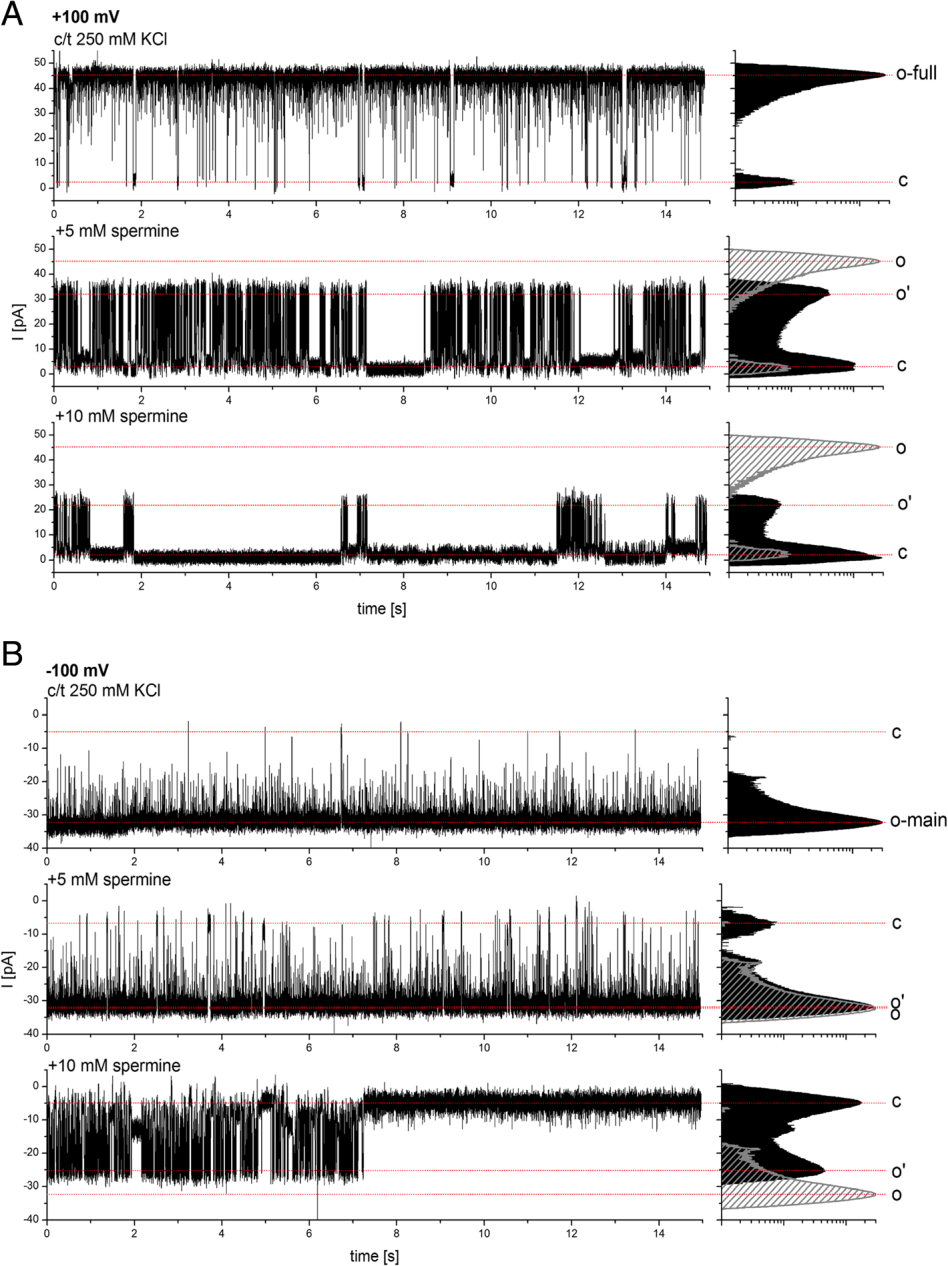
**Channel behavior of PsOep23 in presence of spermine.** Current trace of a single active Oep23 channel at a holding potential of **(A)** +100 and **(B)** -100 mV in control conditions and after addition of 5 and 10 mM of spermine, respectively. The presence of spermine induced a concentration dependent reduction of the open channel conductance (o - > o’) and the open probability, apparent by a more frequent and prolonged likelihood to remain in a closed or blocked state. This block is completely reversible by washing out spermine (data not shown).

Polyamines like spermine, which carries four positive charges at pH 7.0, however reduced both the open channel conductance (o - > o’) by 22-51% and the overall open probability considerably at a concentration of 5 and 10 mM (Figure 5). As the effect was concentration dependent it is most likely that the polyamine directly blocks the pore of the channel or at least hinders the passage of other ions like K^+^. Both sides and all open states of the channel were sensitive to the presence of spermine and the effect was completely reversible after perfusion of the measuring chambers (data not shown). Figure 5 shows a single channel in the fully open state at +100 mV and in main open state at −100 mV. In the presence of spermine both of these conductances are shifted to lower values (o’), meaning that this current reduction is affecting the channel independently of its open conformation.

Spermidine (three positive charges at pH 7.0, Additional file 2: Figure S3) in the same concentration range (5 and 10 mM) did cause neither an obvious change in gating behavior nor in the open probability, but still reduced the overall current through the channel even though to a lesser extent (12-36%). Cadaverine (two positive charges at pH 7.0, channel in open state, o-main, Additional file 3: Figure S4A) and ornithine (one positive charge, channel in open state, o-full, Additional file 3: Figure S4B) did not produce a comparable effect on gating or open probability and only an insignificant change of the overall current (less than 5%).

The pH and the bulk conductivity after addition of up to 10 mM spermidine or spermine did not change significantly (less than 0.2 pH units and 2-3% of the bulk conductivity of control conditions, respectively). Therefore, the pronounced drop of channel conductance had to be attributed to a direct interaction of the compound with the channel protein.

We assume that the triple or quadruple positive charge of spermidine and spermine lead to a binding to the channel’s negative surface charge lining the pore, which might also be responsible for the overall cationic selectivity. Thereby the passage of K^+^ would be gradually impaired and lead to a reduction and finally inhibition of current [29]. If spermine and spermidine can also pass the pore or whether they are hindered for steric or electrostatic reasons is not clear. However, it has been shown that polyamines like spermine and spermidine bind and plug various ion channels, but that blockage is voltage-dependent [30]. Thus a strong depolarization could lead to the passage of spermine and spermidin through PsOEP23 as shown for other ion channels. Since both polyamines have a long linear structure they might be able to pass even through narrow pores [31]. Also the effects of polyamines on the conductance of larger pores like OmpF [26,29] and the translocase of the outer chloroplast membrane Toc75 have been tested [32]. A potential physiological role of spermine flux across the mitochondrial membranes is summarized in [33].

### Circular dichroism spectroscopy

To get an estimation of the overall secondary structure of PsOep23 we measured the circular dichroism spectrum in several detergent and buffer conditions (see material and methods). After analyzing the spectra with four different algorithms the results were consistent with a secondary structure content of 42 ± 10% alpha-helix, 14 ± 5% beta-sheet, 16 ± 5% turns and 27 ± 5% of unordered structures (Table 2). However, since PsOEP23 was purified from inclusion bodies it is possible that the confirmation of the reconstituted protein deviates from the native one even under different reconstitution conditions. Nevertheless, these data indicate that *in vitro* reconstituted PsOep23 channel is mostly formed by amphiphilic helices.

**Table 2:** **Results of CD spectroscopy of PsOep23**

Buffer conditions*	**Alpha helix**			**Beta strand**			**Turn**			**Random**		
	**[%]**			**[%]**			**[%]**			**[%]**		
**1**	39	±	13	15	±	4	20	±	9	26	±	7
**2**	35	±	9	18	±	5	17	±	5	29	±	4
**3**	43	±	6	12	±	3	18	±	4	26	±	5
**4**	52	±	11	11	±	7	11	±	3	25	±	4
**Average**	42	±	10	14	±	5	16	±	5	27	±	5

Distribution of secondary structure in four different buffer conditions and analyzed by four different algorithms.

*Refer to methods.

## Conclusion

Proteomic analysis of cellular organelles and their sub-compartments are an increasingly rich source to identify and characterize proteins of so far unknown function. The chloroplasts outer envelopes form the direct boundary between the organelle and the surrounding cytosol. Their membrane is extremely rich in polar lipids, but most of their protein components are yet poorly characterized. The outer envelope has not only transport functions for ions, metabolites [20] or proteins [11], but is also crucial for signal transduction between the organelle and the cell [34]. In addition it houses enzymes involved in galactolipid biosynthesis and other biosynthetic pathways (for review see [19]). These multiple and very different biochemical functions make it clear that the plastid outer envelope membrane is not a more or less “static” remnant of the endosymbiotic process, but an active player in the biochemical and genetic network of the plant cell. The physiological function of PsOep23 *in vivo* could not be clarified in this study, because no loss-of-function mutants are available. New techniques like the CRISPR/Cas induced gene modification will enable us in the future to generate Oep23 loss-of-function mutants in Arabidopsis and analyze its molecular and architectural phenotype.

## Methods

### Heterologous expression of PsOep23 and inclusion body purification

The PCR amplified full-length psOep23 was cloned into the pET51b(+) vector (Merck, Germany) containing an N-terminal Strep-tag and C-terminal deca-His-tag using *BamH*I and *Sac*I restriction sites. The pET51b(+)-psOep23 construct was transformed into *Escherichia coli* strain BL21 (DE3). Protein expression was induced in exponentially growing cultures (OD_600_ = 0.4) by adding 0.5 mM IPTG. Growth was continued for 4 h at 37°C resulting in PsOep23 inclusion bodies. Cells were harvested by centrifugation, resuspended in 50 mM Tris–HCl pH 8.0, 200 mM NaCl, 5 mM ß-Mercaptoethanol and lysed by passage through a French press and additional sonification. After pelleting PsOep23 inclusion bodies were washed with detergent buffer (20 mM Tris–HCl pH 7.5, 200 mM NaCl, 1% Desoxycholic acid, 1% Nonidet P-40, 10 mM ß-Mercaptoethanol) followed by Triton X-100 buffer (20 mM Tris–HCl pH 7.5, 0.5% Triton X-100, 5 mM ß-Mercaptoethanol) and Tris buffer (20 mM Tris–HCl pH 8.0, 10 mM DTT).

### Solubilization and purification of PsOep23 inclusion bodies for liposome reconstitution

PsOep23 inclusion bodies were solubilized in 50 mM Tris–HCl pH 8.0, 100 mM NaCl, 8 M urea for 1 h at room temperature. Insoluble material was removed by centrifugation at 20,000 g for 15 min and supernatant containing PsOep23 (equiv. to 150 μg protein) was further purified using a 5 ml HisTrap HP column (GE Healthcare, Germany). Bound PsOep23 was washed with five column volumes (CV) of 50 mM Tris–HCl pH 8.0, 100 mM NaCl, 8 M urea buffer containing 35 mM imidazole. Elution was performed by raising the imidazole concentration to 500 mM.

Fractions enriched in PsOep23 were diluted 20-fold in 100 mM Tris–HCl pH 8.0, 150 mM NaCl, 1 mM EDTA, 1% Triton X-100 and further purified using Strep-Tactin affinity matrix. In brief, the protein was allowed to bind to Strep-Tactin Sepharose (iba, Germany) overnight at 4°C followed by washing with 100 mM Tris–HCl pH 8.0, 150 mM NaCl, 1 mM EDTA, 0.1% CHAPS. Protein elution was achieved by 2.5 mM desthiobiotin (iba) at 4°C for 30 min.

In a second approach nickel affinity purified PsOep23 was subjected to buffer exchange in 20 mM sodium phosphate pH 7.0, 8 M urea using Amicon centrifugal filters (MWCO 10 kDa, Merck). The PsOep23 sample was loaded onto a HiTrap Q FF column (GE Healthcare) and flow-through was collected.

### Solubilization of PsOep23 and refolding for circular dichroism

Inclusion bodies of PsOep23 were solubilized in Buffer A (10 mM sodium phosphate pH 7.5, 120 mM SDS) containing 150 mM or 400 mM NaF and subsequently diluted 1:2 in the same buffers containing 3 M 2-methyl-2,4-pentandiol instead of SDS (buffer condition 1 and 2, respectively, adapted from [35]). PsOep23 was refolded for 5 days at room temperature.

Furthermore, aliquotes of PsOep23 solubilized in Buffer A and 150 mM NaF were mixed 1:2 in the same buffer substituted with 120 mM DDM (n-Dodecyl β-D-maltoside) for SDS (buffer condition 3). Refolding of PsOep23 was allowed by dialysis against Buffer A and 2 mM DDM at room temperature.

### Overexpression and purification of soluble PsOep23 for circular dichroism

To obtain soluble PsOep23 *E.coli* BL21 (DE3) harbouring pET51b(+)-psOep23 were induced as described in section 2.1 and shifted to 18°C overnight. Bacteria were washed and resuspended in Buffer B (20 mM sodium phosphate, pH 7.5, 50 mM NaF, 5% glycerol), subjected to French press followed by sonification. After pelleting soluble PsOep23 was purified on a 5 ml HisTrap HP column and washed with Buffer B containing 35 mM imidazole. Bound PsOep23 was eluted by an increase of imidazole to 500 mM. PsOep23 was further purified using Strep-Tactin affinity chromatography. Binding of PsOep23 was allowed for 30 min at 4°C to the Strep-Tactin Sepharose. The matrix was washed with Buffer B and protein elution was performed using 2.5 mM desthiobiotin at 4°C for 30 min. Purified PsOep23 was subsequently dialyzed overnight against Buffer B at 4°C (buffer condition 4).

### Preparation of electrolytes and stock solutions

Cadaverine, Spermidine and Spermine were prepared as 250 mM stock solutions in 1 M Mops/HCl, adenine and thymine as stocks of 150 mM in 1 M KOH, ornithine and malic acid at 1 M in 500 mM Mops/Tris and oxaloacetic acid at 500 mM in 500 mM Mops/Tris. To exclude any indirect effect the pH and the bulk conductivity of the electrolyte were measured after addition of the respective compound. Due to strong alkaline properties of some of these compounds the electrolyte was buffered with 50 mM of Mops/Tris pH 7.0 to guarantee a pH shift of less than 0.2 units and only in the case of adenine and thymine of up to +0.7 units. To ensure that a possible change in channel conductance is due to the compound itself and not to any indirect side-effect bulk conductivity of the electrolyte was measured before and after addition, which was not affected by more than 3%.

Activity coefficients for NaCl and KCl electrolyte concentrations used were interpolated from the values given in CRC Handbook of Chemistry and Physics [36].

### Proteoliposome preparation

Lipid stock solution in chloroform (100 mg/ml, L-alpha-PC, soybean, Larodan, Sweden) was dried under a nitrogen stream for 30 min and dissolved and diluted to a final concentration of 5 mg/ml in buffer containing 80 mM Mega 9, 150 mM NaCl, 10 mM Mops/Tris pH 7.0. Purified PsOep23 was diluted with solubilized lipids by 1:3, mixed with a pipette and incubated on ice for 30 min before being dialysed against 150 mM NaCl, 10 mM Mops/Tris pH 7.0 at 4°C overnight.

In a second approach the flow-through from a cation exchange column containing PsOep23 was diluted 1:2 with solubilized lipid solution, incubated at room temperature and dialysed against 150 mM NaCl, 20 mM sodium phosphate pH7.5 at room temperature overnight and again for 6 h at 4°C.

### Flotation assay

Liposome-associated and liposome free proteins were separated by flotation through a step gradient of Nycodenz medium (Axis Shield, Norway) in 20 mM Hepes/NaOH pH 7.5, 150 mM NaCl. The sample was adjusted to a Nycodenz concentration of 20% (0.5 ml) and overlaid with a step gradient of 2 ml 15%, 1 ml 10% and 0.5 ml 0% Nycodenz. It was centrifuged at 217,000 g for 2 h at 4°C. Fractions of 0.5 ml were collected and precipitated with trichloroacetic acid and the pellet was washed with ice-cold acetone before dried and resuspended in Laemmli buffer. Samples were analyzed by SDS-PAGE (12.5% gel), blotted onto a PVDF membrane and imuno-decorated with a polyclonal Oep23 antibody, which was raised against the heterologously expressed full-length protein from pea. The blotting membrane was eventually stained with Coomassie brilliant blue to check for overall purity of the fractions (Additional file 4: Figure S1).

### Electrophysiology

Electrophysiological measurements were performed as described in [37]. In brief, planar lipid bilayers were produced by the painting technique. A solution of 50 mg/ml L-alpha-PC (soybean, type IV-S, Sigma, Germany) in n-decane (Sigma) was applied to a hole (50–100 μm diameter) in a Teflon septum, separating two bath chambers (volume of 3 ml each). Both chambers were equipped with magnetic stirrers. By continuously lowering and re-raising of the solution level, the lipid layer across the hole was gradually thinned out until a stable bilayer was formed. This formation was monitored optically. The resulting bilayers had a typical resistance of >100 GΩ. The noise was 2.9 pA (root mean square) at 3 kHz bandwidth. As an osmotic gradient of a channel permeant solute and a channel in the open state are prerequisites for fusion of proteoliposomes with the bilayer, the initial electrolyte conditions were cis and trans 20 and 250 mM KCl, 10 mM Mops/Tris, pH 7.0. Proteoliposomes were then added to the trans chamber directly below the bilayer to cause a slow flow of proteoliposomes along the bilayer surface. For subsequent experiments both chambers were perfused to the required conditions by a 20-fold volume within a period of 1 min. The Ag/AgCl electrodes were connected to the chambers through 2 M KCl, 1.5% agar salt bridges. The electrode of the cis compartment was directly connected to the head stage of a Patch clamp amplifier (EPC10 USB, Heka). Reported membrane potentials are always referred to the cis compartment. Current traces were recorded at a frequency of 10 kHz and pre-filtered with a 3 kHz Bessel filter. For analysis, HEKA data files (.dat) were converted to Axon binary files (.abf) with the help of the ABF File Utility Software (Version 2.1.76) and further processed in ClampFit (Molecular Devices). Diagrams were prepared by using Origin (OriginLab). The gating frequency was determined by the threshold search algorithm of ClampFit with the base line set to the main open current state and a threshold just outside the noise level of 15 s current traces at +/−100 mV, respectively. Events below a minimum duration of 1 ms were rejected. The I/V curve was generated from 16 individual bilayers. Each individual data point corresponds to the peak value of a current histogram from traces at the respective voltage. Values were classified and averaged; the error corresponds to the standard deviation. The selectivity of Oep23 was determined in either asymmetric KCl electrolytes (20 and 250 mM) or in bi-ionic conditions (cis 242 mM NaCl, trans 250 mM KCl). The measured values of the reversal potential (E_rev_) were corrected by liquid junction potentials and calculated with the Clampex software (Molecular Devices). All calculations considered the activity rather than concentration of the electrolyte.

### Circular dichroism spectroscopy

For the determination of protein concentration UV spectra from 220–400 nm of a 10–20 times diluted sample in 10 mM sodium phosphate pH 7.5 a were taken (Specord 40, Analytik Jena, Germany) and the absorbance at 280 nm, subtracted by the value at 320 nm to compensate for offset and scattering, was converted into molar concentrations by using Lambert-Beer’s law with a primary sequence derived extinction coefficient from ProtParam. Concentrations usually were in the range of 0.8-3.7 μM or 21–100 μg/ml.

Circular dichroism spectra were measured with a Jasco J-810 spectropolarimeter in a 1 mm quartz cuvette from 178–260 nm and data corresponded to an average of 10–15 repeated scans and subtracted by the reference spectrum of the buffer alone. Data were analysed from 185–240 nm or 195–240 nm depending on the photomultiplier voltage (HT-value) in the shorter wavelength range using four different algorithms: Raussen’s method, CDSSTR (set 3/4) SELCON (set 3/4) and CONTIN (set 3/4) [38,39].

### Availability of supporting data

The data supporting the results of this article are included within the article.

## Additional files

Additional file 1: Figure S2. Sequence alignment of PsOep23 and AtOep23 using ClustalW (www.ebi.ac.uk/Tools/msa/clustalw2/). The DUF1990 domain is shaded in grey and the identified peptide after mass spectrometry analysis is indicated by a black line. Additional file 2: Figure S3. Channel behavior of PsOep23 in presence of spermidine. Current trace of a single active Oep23 channel at a holding potential of **(A)** +100 and **(B)** -100 mV in control conditions and after addition of 5 and 10 mM of spermidine, respectively. Spermidine also induces a concentration dependent reduction of the open channel conductance but to a smaller extend compared to spermine (see Figure 5). A change of gating behavior and open probability was not detected at these concentrations. The effect is also completely reversible by wash out (data not shown). Additional file 3: Figure S4. Channel behavior of PsOep23 in presence of cadaverine or ornithine.Current trace of a single active Oep23 channel at a holding potential of +100 and −100 mV in control conditions and after addition of 5 and 10 mM of cadaverine **(A)** and 2 and 10 mM ornithine **(B)**, respectively. Neither cadaverine nor ornithine induced a change in the open probability or gating behavior. The slight change in open channel conductance is insignificant. Additional file 4: Figure S1. Reconstitution of PsOep23 into liposomes. After reconstitution of purified PsOep23 into liposomes of L-alpha phosphatidylcholine Oep23 floated in a step gradient of Nycodenz (0, 10, 15, 20%) to lower densities indicative for a successful incorporation into the liposome bilayer. The lower panel shows an immunoblot demonstrating the presence of PsOep23 in the different fractions of the gradient.

## Abbreviations

DDM: n-Dodecyl β-D-maltoside
GHK: Goldmann-Hodgkin-Katz equation
IPTG: Isopropyl β-D-1-thiogalactopyranoside
Oep: Outer envelope protein
Omp: Outer membrane protein
PC: Phosphatidylcholine
Sam: Sorting assembly machinery
SDS: Sodium dodecyl sulfate
Toc: Translocon of the outer chloroplast membrane
VDAC: Voltage dependent anion channel
