# Supplementary material for: Oep23 forms an ion channel in the chloroplast outer envelope
Source: BMC Plant Biol. 2015 Feb 12;15:47. doi: 10.1186/s12870-015-0445-1 (PMC4331141; doi:10.1186/s12870-015-0445-1)
Supplement: Additional file 1: Figure S2. — Sequence alignment of PsOep23 and AtOep23 using ClustalW (www.ebi.ac.uk/Tools/msa/clustalw2/). The DUF1990 domain is shaded in grey and the identified peptide after mass spectrometry analysis is indicated by a black line. [file 12870_2015_445_MOESM1_ESM.pdf]

```
PsOep23 MVFLSWVRPTTQDQNTCINKSGTFNYDDRYKGATAKSLSSLQQDKALSNDGFLLNNARVL 60
AtOep23 MVFLSWGRPSSEQQQQVINKTGTFNYDNKYRGVSSRSIAKLKEDSEIDKDGFLINHARVL 60
        ****** **::::*:  ***:******::*:*.:::*::.*::*. :.:****:*:****

PsOep23 IGNGIDTFEKGKTALRTWRHFGMNWAFVDPETPIQQGAKFCICVKEFLPWLMMPLQVVYV 120
AtOep23 VGSGRESYEKGKKALQNWKHFGMDWAFVDPATPVETGKKFCICVKEVLPWVMLPLQVVYV 120
        :*.* :::****.**:.*:****:****** **:: * ********.***:*:*******

PsOep23 NETKTTKNRGASFGFGSGTLQGHLLAGEERFSIEIDENNQVWYEILSFSKPAHVLSFVGY 180
AtOep23 DESRKSRKGPAHFGYGSGTLQGHLLAGEEKFSIELDGNGEVWYEITSFSKPAHFLSFLGY 180
        :*::.:::  * **:**************:****:* *.:***** *******.***:**

PsOep23 PYVMLRQKYFAHESAKVMLKHINSSKS 207
AtOep23 PYVKLRQKHFARHSSEAVLKHVNAS-- 205
        *** ****:**:.*::.:***:*:*
```
