# Supplementary figures and images for: Oep23 forms an ion channel in the chloroplast outer envelope
Source: BMC Plant Biol. 2015 Feb 12;15:47. doi: 10.1186/s12870-015-0445-1 (PMC4331141; doi:10.1186/s12870-015-0445-1)

**A**

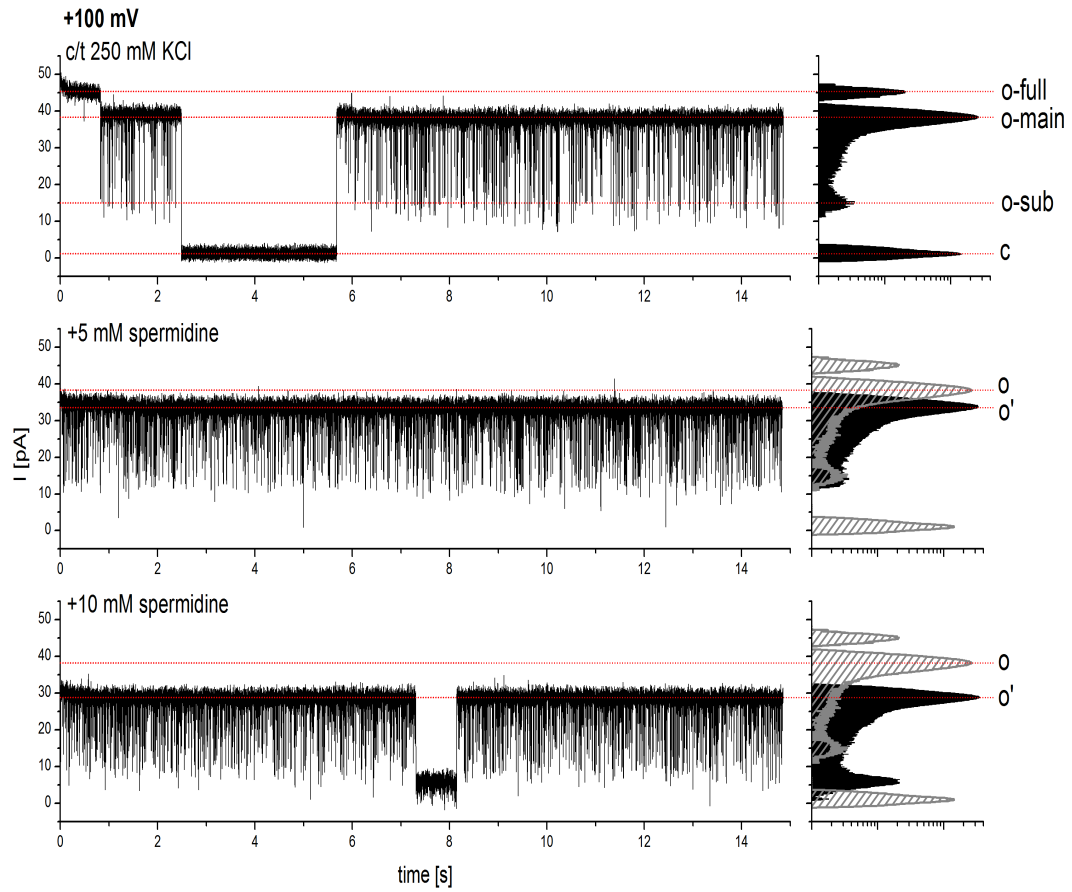

**B**

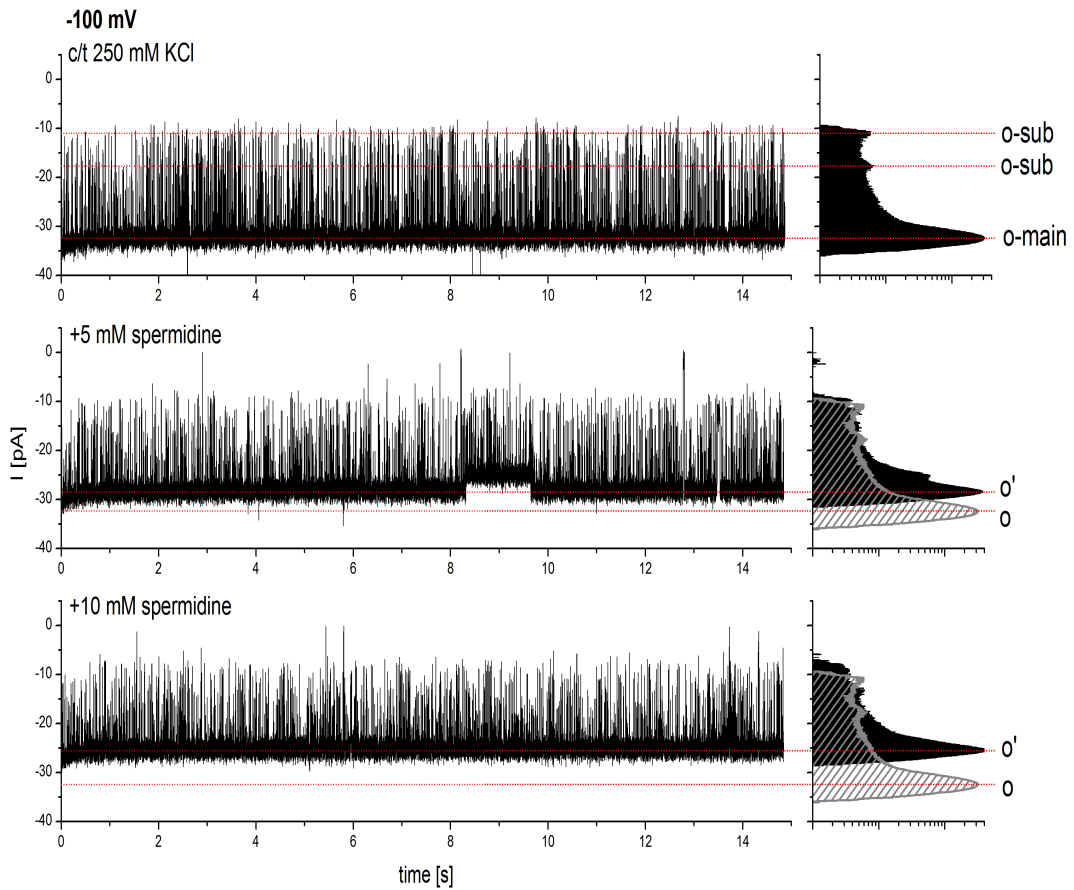

Supplement: Additional file 2: Figure S3. — Channel behavior of PsOep23 in presence of spermidine. Current trace of a single active Oep23 channel at a holding potential of (A) +100 and (B) -100 mV in control conditions and after addition of 5 and 10 mM of spermidine, respectively. Spermidine also induces a concentration dependent reduction of the open channel conductance but to a smaller extend compared to spermine (see Figure 5). A change of gating behavior and open probability was not detected at these concentrations. The effect is also completely reversible by wash out (data not shown). [file 12870_2015_445_MOESM2_ESM.pdf]

A

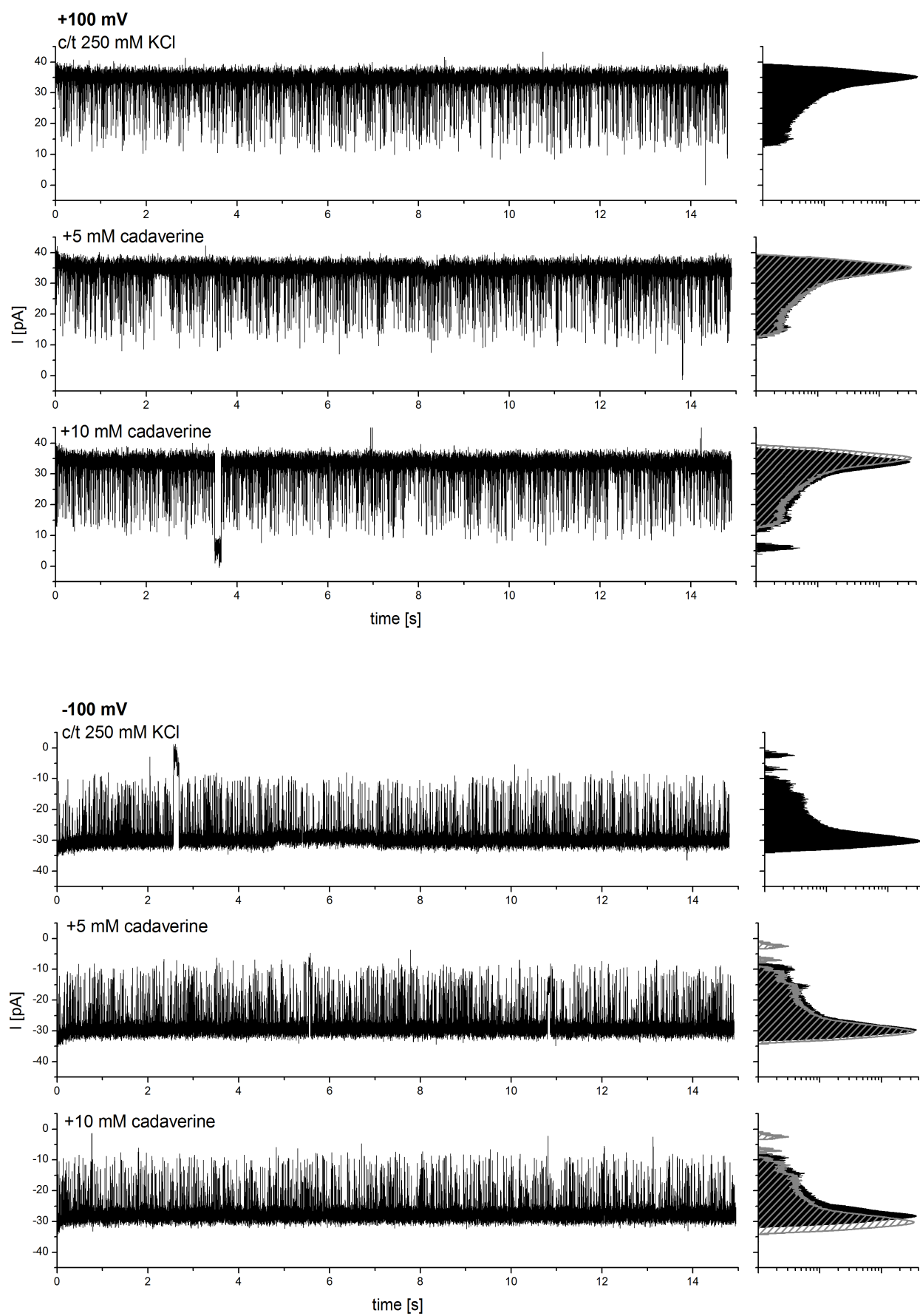

Supplement: Additional file 3: Figure S4. — Channel behavior of PsOep23 in presence of cadaverine or ornithine.Current trace of a single active Oep23 channel at a holding potential of +100 and −100 mV in control conditions and after addition of 5 and 10 mM of cadaverine (A) and 2 and 10 mM ornithine (B), respectively. Neither cadaverine nor ornithine induced a change in the open probability or gating behavior. The slight change in open channel conductance is insignificant. [file 12870_2015_445_MOESM3_ESM.zip › 1719249340149747_add4.pdf]

**B**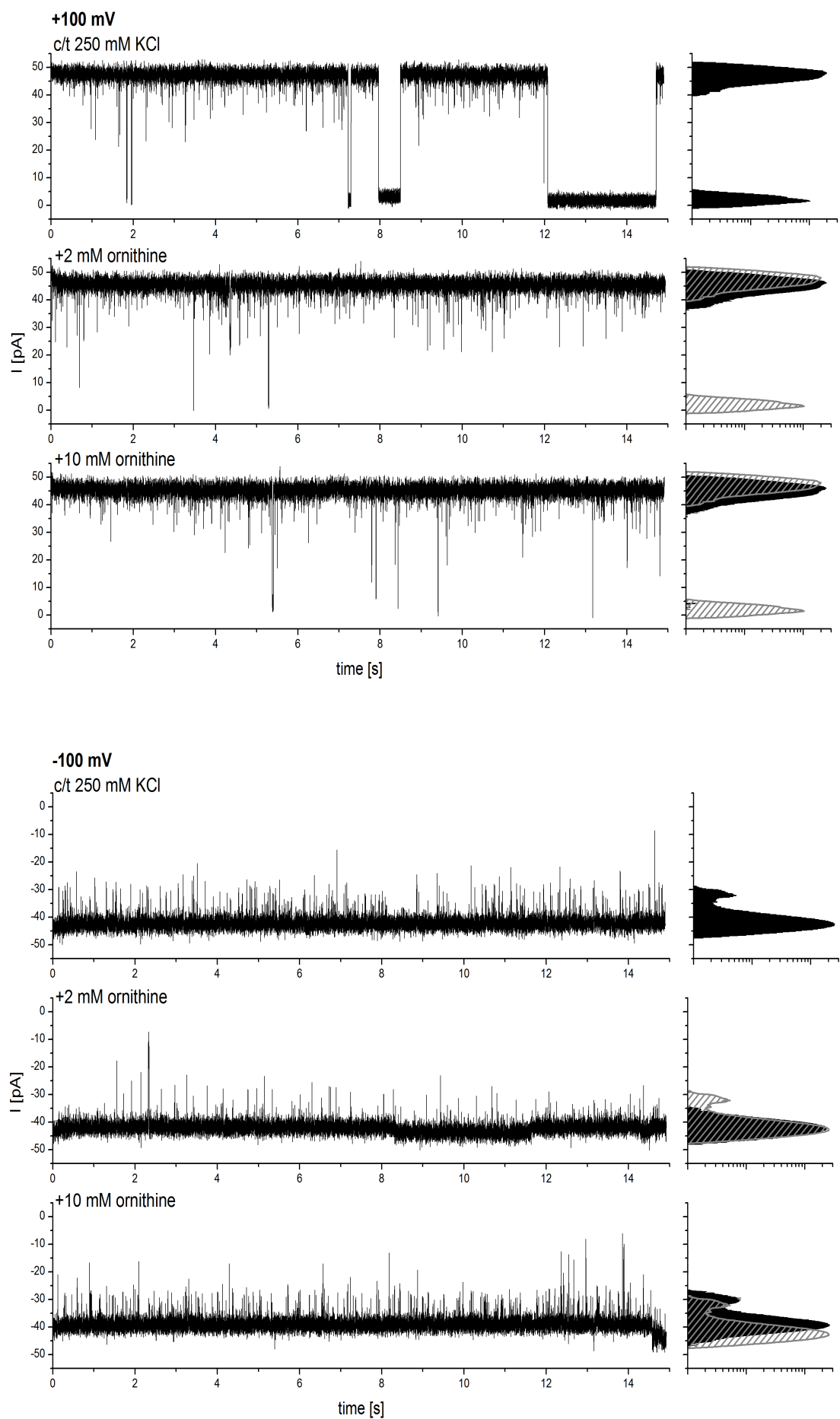

Supplement: Additional file 3: Figure S4. — Channel behavior of PsOep23 in presence of cadaverine or ornithine.Current trace of a single active Oep23 channel at a holding potential of +100 and −100 mV in control conditions and after addition of 5 and 10 mM of cadaverine (A) and 2 and 10 mM ornithine (B), respectively. Neither cadaverine nor ornithine induced a change in the open probability or gating behavior. The slight change in open channel conductance is insignificant. [file 12870_2015_445_MOESM3_ESM.zip › 1719249340149747_add5.pdf]

**Nycodenz [%]**

|   |    |    |    |
|---|----|----|----|
| 0 | 10 | 15 | 20 |
|---|----|----|----|

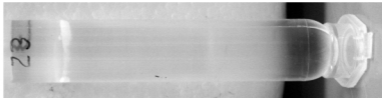

**anti OEP23**

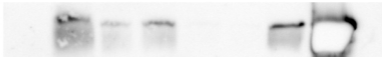

Supplement: Additional file 4: Figure S1. — Reconstitution of PsOep23 into liposomes. After reconstitution of purified PsOep23 into liposomes of L-alpha phosphatidylcholine Oep23 floated in a step gradient of Nycodenz (0, 10, 15, 20%) to lower densities indicative for a successful incorporation into the liposome bilayer. The lower panel shows an immunoblot demonstrating the presence of PsOep23 in the different fractions of the gradient. [file 12870_2015_445_MOESM4_ESM.pdf]
